# Book Notices

**Published:** 1866-10

**Authors:** 


### EDITORIAL.

### BOOK NOTICES.

*Medical Electricity: Embracing Electro-Physiology and Electricity as a Therapeutic, with Special Reference to Practical Medicine, showing the most approved Apparatus, Methods and Rules for the Medical Uses of Electricity in the Treatment of Nervous Diseases.* By ALFRED C. GARRATT, M.D., Fellow of the Massachusetts Medical Society, Member of the American Medical Association. Third edition, revised and illustrated. Philadelphia: J. B. Lippincott & Co. 1866. Pp. 1103.

What shall we say of an author who almost at the outset of his work requires of his readers the comprehension of such terms as *anelectrotonus* and *katelectrotonus!* Perhaps Dr. Garratt chooses as a style appropriate to his matter one which involves the subject in all the clouds that can be summoned from the "vasty deep" of chaotic thought. At any rate he has presumed most unwarrantably upon the patience of his professional brethren in offering them a volume so ponderous as this third edition of his book. It is in itself a thunderstorm, a hurricane of words, furrowed with gleaming tracks of italic letters, which may be supposed to symbolize the lightning flashes upon the darkness of the tempest. And yet the volume is full of good stuff. It presents facts which the student can only with difficulty obtain from the original sources. It descends to all the minutiæ of description which are so important in text-books for the use of beginners. It is as complete a discussion of the subject of Medical Electricity as can be found in the English language. It acquaints us, in the person of the author, with a genial, agreeable, enthusiastic student of Nature.

It would be a great work and a treasury of learning such as could not be dispensed with, were it not for the flood of garrulity which pours through every paragraph to the extent of eleven hundred solid pages. One might consent to overlook the minor errors due to the carelessness of the proof-reader, but not such as affect the substance and the order of the work itself. Had we time and space we could amuse our readers with a few illustrative passages by way of example of our author's quality, but that is out of the question. Whoever cares for such things must read for himself in the original volume.

We regret that we must speak thus of a work from which we had hoped for so much instruction. Of information there is certainly enough to satisfy any reasonable mortal, but it is not in a form to benefit the members of a busy profession. We need a monograph which in method and in point shall match the lectures of Radcliffe on Pain, Epilepsy and Paralysis. If some one will take Dr. Garratt his book, and will shake out the nonsense between its covers, perhaps the residuum may constitute the object of our desires.

---

*A Treatise on the Origin, Nature, Prevention and Treatment of Asiatic Cholera.* By JOHN C. PETERS, M.D. New York: D. Van Nostrand, 192 Broadway. 1866.

We have read this monograph with much pleasure. An agreeable style does wonders for a dull subject. For the first time we have been interested by the chapter which forms the dreary introduction to all other books on cholera—the chapter which is devoted to the history of the origin of the disease. Thanks to Dr. Peters, we have waded through the filth of Bigginuggar, Ramieseweram, of Jessore, Mysore, Congeiveram and the feast of Kurbar-Bariam without losing all stomach for the remainder of the book. This we consider a victory of no small importance to the author. The chapter on the course and distribution of cholera presents an array of facts and arguments in proof of the portability and communicability of the germs of cholera which may be considered as decisive. It will be difficult for any one hereafter to entertain the miasmatic or atmospheric-wave theory of the causation of the disease. It appears certain that the victim of cholera throws off, probably through the medium of the intestinal dejections, innumerable germs which are transported in a manner analogous to the distribution of the seeds of plants. The causes which favor the germination and reproduction of the one class of germs, are strictly analogous to those favorable to the growth of the other. The experience of our own community during the present visitation of cholera, fully illustrates this proposition. Warmth, moisture, filth and physical predisposition afford the soil upon which cholera flourishes and spreads. When these elements are withdrawn, the prevalence of the disease is reduced to a minimum, and it becomes extinct, precisely as the seed of the sower springs not up when cast upon the dry and stony rock. The author states his belief concerning the propagation of cholera as follows:

The communicability of the disease does not correspond with the time when the dejections are voided; but is only developed a few days subsequently, and seems to be exhausted at the end of fifteen to twenty-one days. This peculiarity has been traced to the fact that the rice-water discharges only become poisonous after a while; for the first few days (hours?) they are inocuous; then, as decomposition proceeds, they become morbific, and capable of reproducing the peculiar disease of which they were the product. * * * After a few days more, when decomposition has reached a farther stage, the contagious property of the evacuations cease. These great facts account for the impunity with which careful and cleanly persons may wait upon those sick with cholera; for the mysterious and sudden outbreak of the disease, and for its equally sudden subsidence.

These points have been proved, in the following ingenious way: Pieces of filtering paper, soaked in the rice-water discharge have been given to mice, mixed with their food, and it was found that papers steeped in the very recent, and others dipped in the older discharges, proved alike harmless. But of the thirty-four mice that ate paper impregnated with excretæ of an intermediate date, thirty became sick, and twelve died; while the symptoms and appearances noticed after death, are declared to have been similar to those that are proper to cholera as it was seen in the human subject.

The section which is devoted to the theory of cholera reviews the various pin-hole theories of the disease which have been from time to time presented to the profession. We are puzzled to know why that one which the author seems inclined to adopt for his own guidance, should be designated by the title of *Physiological Theory.* According to this notion the ten or twelve quarts of fluid which are daily exuded by the mucous surface of the stomach and intestines, are in cholera no longer reabsorbed, but are expelled from the body. There is certainly nothing worthy of the term *Physiological* in such a process. The "gland theory" which was advanced by Dr. Isaac Hayes, is now maintained by very few individuals. The "elimination theory" has already been fairly presented to the profession. The "paralysis theory" attributes the disease to a paralysis of the vessels and nerves of absorption, and requires the use of tonics and stimulants, such as strychnia, camphor, alcohol, quinine, etc. The "first spasm theory" supposes that the blood into which the cholera-poison has entered, is irritant in its nature, and that it excites spasmodic contraction of the muscular walls of the minute pulmonary arteries. From this proceed all the subsequent phenomena of the disease. Bell and Braithwaite have adopted a modification of this idea, which Dr. Peters calls the "second spasm theory," but that does not cover the whole ground. "It seems perfectly transparent that Johnson, Bell and Braithwaite have mistaken the secondary spasm of the vascular system in general, for a primary disorder." Dr. George Hamilton's theory that "profound passive congestion" is the root of evil, stands last on the list, and cannot be considered as anything but an unsubstantial shadow of a theory. The author himself advances nothing original as a substitute for the various theories which have been enumerated. Perhaps this should not be required in a work which is intended to represent other men's ideas rather than his own.

Under the head of prevention of cholera, prominence is given to quarantine, cleanliness and disinfection. It is sufficient to say that Dr. Peters believes in a long continued and rigid quarantine.

The section on treatment is little more than a catalogue of the various remedies which have been used by all classes of doctors—homœopathic included. Having enjoyed peculiar advantages for the observation of every variety of treatment, the author is enabled to speak with authority, and some of his revelations are both curious and interesting. For example, we learn that "in 1832, the Edinburgh homœopathists used over five gallons of the tincture of camphor." Those gentlemen surely could not have belonged to the Hahnemaniac wing of the faction. We are, furthermore, informed that the tincture of camphor used by the homœopathists, is nearly five times as strong as the one in ordinary use. Kurtz and other homœopathists use teaspoonful doses of a solution of one grain of tartar emetic, in one or two ounces of water. Very good for the patient, but rather bad for homœopathy. In cases of collapse, "Fleischman and Tessier, who have had the largest homœopathic hospital experience, say that homœopathy is comparatively powerless." Tessier says, "it seems fair to treat the black and ataxic forms of cholera in the usual manner, inasmuch as homœopathy fails completely in both of these varieties." Among reputable practitioners, the treatment by sulphuric acid is said to be the fashionable mode at present. Dr. P., however, gives the preference to sulphate of iron or iron-alum. Stimulants and opiates should be used very sparingly and in small doses.

The book is beautifully printed on tinted paper, and forms a work which does credit to all who have been concerned in its production.

---

*The Principles of Surgery.* By JAMES SYME, F.R.S., Surgeon in Ordinary to the Queen in Scotland, Prof. of Surgery, etc. To which are appended his Treatises on the "Diseases of the Rectum," "Stricture of the Urethra and Fistula in Perineo," "The Excision of Diseased Joints," and numerous additional contributions to the Pathology and Practice of Surgery. Edited by his former pupil, DONALD MACLEAN, M. D., F. R. C. S. E., Prof. of the Institutes of Medicine and Lecturer on Clinical Surgery, Queen's University, Canada. Philadelphia: J. B. Lippincott & Co. 1866.

An edition of Syme's Surgery was issued in this country a few years since, by R. S. Newton, a Professor in the Eclectic School, "Cancer Doctor," etc., etc. This edition not being accepted by the members of the regular profession, of course added nothing to Prof. Syme's reputation in this country, and the profession is under obligations to Prof. Maclean for a correct edition of the work; while the distinguished Edinburgh Professor has reason to congratulate himself that one of his former pupils has redeemed it from the disgrace into which it had fallen at the hands of the "Eclectics."

The largest part of the work before us is devoted to the "Principles of Surgery;" a considerable portion, however, is given to special treatises upon important subjects, namely, "Diseases of the Rectum," "Stricture of the Urethra and Fistula in Perineo," "The Excision of Diseased Joints;" and over 150 pages are devoted to "Clinical Observations;" also, a report of three cases of excision of the scapula, and one case of excision of the tongue.

Every page of the work abounds in matter of interest, but it is these special treatises, etc., which render the work so particularly valuable; and for these alone, no student or practitioner of surgery, can regret the amount expended for the book. Its mechanical execution is satisfactory.

For sale by W. B. Keen & Co. Price $7.

---

*On Spermatorrhœa: Its Causes, Symptomatology, Pathology, Prognosis, Diagnosis and Treatment.* By ROBERTS BARTHOLOW A. M., M. D., etc., etc. New York: Wm. Wood & Co.

A very neat and sufficient monograph. The author thus states his reasons for writing upon this subject: "I think it is a reproach to our profession that this subject has been permitted, in a measure by our own indifference, to pass into the hands of the unscrupulous pretenders, whose suggestive publications are amongst the crying evils of our time. Because the subject is disagreeable, and to a certain degree disreputable, competent physicians are loth to be concerned with it. The same unnecessary fastidiousness causes the treatment of this malady to be avoided in private practice; and the unfortunate patients, thus precluded from obtaining intelligent advice, fall into the hands of advertising specialists, who excite their worst apprehensions for a mercenary purpose. For this reason, and to obviate the sad consequences which result from spermatorrhœa, it is our duty to exert our best efforts in behalf of those afflicted with this malady. We should endeavor to attain to correct views of its pathology, and apply our knowledge to its cure—if for no other reason, for the good of our species."

For sale by W. B. Keen & Co.

---

*A Manual of the Principles of Surgery, based on Pathology, for Students.* By Wm. Canniff, Licentiate of the Medical Board of Upper Canada, M. D. of the University of New York, M. R. C. S. England, etc., etc. Philadelphia: Lindsay & Blakiston. 1866.

Having carefully looked over this work, we feel prepared to say, that the author has accomplished all that he set out to do. The work will not only prove a useful "Manual for Students," but one of very considerable interest to the practitioner. There are many subjects fully treated of, and the various stages of the malady spoken of in detail, which are only cursorily dwelt upon by professors in the lecture-room, or by the standard authors on surgery. It is this fact more particularly than any other, perhaps, which should insure for the book a cordial welcome to the medical library.

The divisions of the work are as follows:

1st. Inflammation and Diseases arising out of Inflammation.

2d. The Healing Process and Diseases of the Healing Process.

3d. External Injuries—Contusions and Wounds.

4th. Diseases of certain Tissues, Bones, Joints, (including Fractures and Dislocations,) Arteries and Veins.

5th. Morbid Growths.

The articles on "External Injuries," under which division the author treats of "Lacerated, Contused, Punctured, Gunshot and Poisoned Wounds," are concise and to the point, plainly showing to the reader that the writer has very much of practical as well as theoretical schooling. We hope that the book may meet with the hearty welcome which its positive merits seem to warrant, and that shortly it will be followed by other efforts from our Canadian medical friends, since heretofore there have been few books upon this and kindred subjects emanating from that quarter.

For sale by W. B. Keen & Co. Price $4.50.

---

*The Medical Register of the City of New York, for* 1866. By GUIDO FURMAN, M. D.

A valuable little hand-book, which contains full lists of all the societies, hospitals, charities and members of the medical public in New York.

